# Vascular and Inflammatory Factors in the Pathophysiology of Blast-Induced Brain Injury

**DOI:** 10.3389/fneur.2015.00048

**Published:** 2015-03-16

**Authors:** Gregory A. Elder, Miguel A. Gama Sosa, Rita De Gasperi, James Radford Stone, Dara L. Dickstein, Fatemeh Haghighi, Patrick R. Hof, Stephen T. Ahlers

**Affiliations:** ^1^Neurology Service, James J. Peters Department of Veterans Affairs Medical Center, Bronx, NY, USA; ^2^Department of Psychiatry, Icahn School of Medicine at Mount Sinai, New York, NY, USA; ^3^Department of Neurology, Icahn School of Medicine at Mount Sinai, New York, NY, USA; ^4^Friedman Brain Institute, Icahn School of Medicine at Mount Sinai, New York, NY, USA; ^5^Research and Development Service, James J. Peters Department of Veterans Affairs Medical Center, Bronx, NY, USA; ^6^Department of Radiology and Medical Imaging, University of Virginia, Charlottesville, VA, USA; ^7^Department of Neurosurgery, University of Virginia, Charlottesville, VA, USA; ^8^Fishberg Department of Neuroscience, Icahn School of Medicine at Mount Sinai, New York, NY, USA; ^9^Department of Geriatrics and Palliative Care, Icahn School of Medicine at Mount Sinai, New York, NY, USA; ^10^Department of Neurotrauma, Operational and Undersea Medicine Directorate, Naval Medical Research Center, Silver Spring, MD, USA

**Keywords:** animal models, blast, inflammation, traumatic brain injury, vascular pathology

## Abstract

Blast-related traumatic brain injury (TBI) has received much recent attention because of its frequency in the conflicts in Iraq and Afghanistan. This renewed interest has led to a rapid expansion of clinical and animal studies related to blast. In humans, high-level blast exposure is associated with a prominent hemorrhagic component. In animal models, blast exerts a variety of effects on the nervous system including vascular and inflammatory effects that can be seen with even low-level blast exposures which produce minimal or no neuronal pathology. Acutely, blast exposure in animals causes prominent vasospasm and decreased cerebral blood flow along with blood-brain barrier breakdown and increased vascular permeability. Besides direct effects on the central nervous system, evidence supports a role for a thoracically mediated effect of blast; whereby, pressure waves transmitted through the systemic circulation damage the brain. Chronically, a vascular pathology has been observed that is associated with alterations of the vascular extracellular matrix. Sustained microglial and astroglial reactions occur after blast exposure. Markers of a central and peripheral inflammatory response are found for sustained periods after blast injury and include elevation of inflammatory cytokines and other inflammatory mediators. At low levels of blast exposure, a microvascular pathology has been observed in the presence of an otherwise normal brain parenchyma, suggesting that the vasculature may be selectively vulnerable to blast injury. Chronic immune activation in brain following vascular injury may lead to neurobehavioral changes in the absence of direct neuronal pathology. Strategies aimed at preventing or reversing vascular damage or modulating the immune response may improve the chronic neuropsychiatric symptoms associated with blast-related TBI.

## Introduction

It was first recognized during world war (WW) I that blast exposure can be associated with psychological and neurological symptoms reminiscent of both the post-concussion syndrome and what would now be called post-traumatic stress disorder (PTSD) (1). While an uncommon cause of traumatic brain injury (TBI) in civilian life (2), blast-related TBI has been of longstanding interest in military medicine and has recently received wider attention because of its frequency in the military operations in Iraq and Afghanistan (3–7). Estimates are that 10–20% of veterans returning from these conflicts have suffered a TBI with the most frequent cause being blast exposure related to improvised explosive devices (IED) (8, 9).

Initially most attention focused on the moderate to severe end of the TBI spectrum (10), the type of injury that would be recognized in-theater, and the war in Iraq has resulted in the highest number of service-related severe TBIs since the Vietnam era (11). However, what became apparent was that many Iraq and Afghanistan veterans were presenting to veterans affairs (VA) hospitals and other facilities with symptoms suggestive of the residual effects of mild TBIs (mTBIs) that were never recognized prior to discharge. Indeed mTBIs vastly outnumber moderate to severe TBIs in these veterans (8, 9).

Interest in how blast affects the nervous system has led to a rapid expansion of clinical as well as animal studies. Animal studies show that blast pressure waves are transmitted through the brain and exert a variety of biochemical, pathological, and pathophysiological effects (12, 13). However, questions remain concerning how these pathologies lead to blast-related symptoms as well as how, or even if, the pathologies are related to one another. Blast-related vascular and inflammatory effects are well-established (14) and occur with even low-level exposures that produce minimal or no detectable neuronal pathology. Here, we review the evidence that blast exposure causes acute and chronic vascular as well as inflammatory effects and how these effects may play a role in the pathophysiology of chronic blast-related brain injury.

## Vascular Pathology in Blast-Induced Brain Injury

Vascular pathology is a well-recognized component of non-blast TBI in humans and animals (15). Few human cases of acute blast exposure have come to autopsy and many that have sustained such severe injuries that they died within a few days of injury (16). The most prominent features in two cases from WWI studied by Mott (17) were punctate hemorrhages in subcortical gray and white matter regions. Cohen and Biskind (18) identified nine cases from WWII in the archives of the US Armed Forces Institute of Pathology, all of whom died within 5 days of injury. These cases also exhibited a prominent hemorrhagic component with diffuse leptomeningeal bleeding, intracerebral clots, and multifocal hemorrhages in white matter. More recent observations in-theater suggest that edema, intracranial hemorrhage, and vasospasm are prominent features of severe blast injury in the current conflicts (19, 20). In a review of neurosurgical consultations on soldiers transferred from Iraq to the National Navel Medical Center, Armonda et al. (19) found that vasospasm was present in nearly half of those undergoing angiography as part of a diagnostic evaluation for acute neurotrauma. Vasospasm was often prolonged averaging 14 days in duration with some cases persisting up to 30 days. Pseudoaneurysm formation as well as hemorrhage was often seen in association with vasospasm, which generally predicted a worse outcome (19). In a review of UK fatalities in Afghanistan, some form of hemorrhage was implicated as the proximal cause of death in most cases (21). Thus high-pressure blast waves can cause extensive CNS injury in the context of severe TBI in humans with a strong hemorrhagic component.

The role of vascular pathology in human cases of mTBI is less clear. Cases of mTBI rarely come to autopsy, and little is generally known about the pathology of mTBI especially in the acute phase (22). Only a few cases of blast-related TBI have been studied in the chronic phase (23–26). Several of these exhibited a chronic traumatic encephalopathy-like picture with a prominent tauopathy that included perivascular tau deposition (23–25). Ryu et al. (26) have also described amyloid precursor protein-positive axonal swellings typical of the diffuse axonal injury that can be seen long after non-blast TBI. In white matter, these abnormalities occurred primarily in a perivascular distribution (26).

Due to the lack of human data, most of our knowledge of how blast exposure affects the nervous system comes from animal studies. Animals have been exposed to various forms of blast ranging from direct exposure to live explosives to more commonly controlled blast waves produced by compressed-air generators with rats being most often used (3, 13). Approaches have varied. Some studies have subjected animals to whole body blast, while others attempting to isolate effects on the CNS have selectively exposed the head or utilized various forms of shielding to protect the body. Various pathological effects have followed blast overpressure injuries in these models (3, 13). These effects have in turn been associated with biochemical and pathophysiological changes as well as behavioral phenotypes. Issues concerning the use of these models have been discussed in several recent reviews (12, 27–29).

Blast-related vascular effects in animals are summarized in Tables 1 and 2, where studies are divided into those using whole body blast (Table 1) and those that have isolated exposure to the head or body (Table 2). As in humans, acute high-level blast exposure has a prominent hemorrhagic component in species including rats, mice, rabbits, ferrets, and goats (30–37). Varying degrees of subdural, subarachnoid, and intracerebral hemorrhage are visible grossly and microscopically along with edema and diffuse vascular congestion on the brain surface. Effects seem similar whether exposures under shock tube conditions or live explosives are used with a clear correlation between injury severity and blast overpressure levels or distance from the detonation in the case of live explosives.

**Table 1:** **Vascular and inflammatory changes in the nervous system following whole-body blast exposure in animal models**.

Species	Blast exposure	Vascular effects	Inflammatory effects	Reference
Rat	110 kg TNT, exposed in a simulated bunker		Increased macrophages/microglia in pineal gland based on OX-42, OX-18, OX-6, and ED1 staining, increases at 7, 14, and 21 days after blast returning to normal by 28 days, most in perivascular locations	(38)
Pig	Howitzer (9 and 30 kPa, peak overpressure, durations: 2.4 to 5.1 ms), bazooka (41 kPa, 1.9 ms), and automatic rifle (21–23 kPa, 0.7–1.3 ms) in open field or an enclosure	3 and 7 days after three blast exposures, small parenchymal and subarachnoid hemorrhages predominately in occipital lobe, cerebellum, and medulla oblongata in animals exposed to bazooka, 30 kPa Howitzer, and automatic rifle in an open field, hemorrhages not observed in animals exposed to 9 kPa Howitzer or automatic rifle in enclosure		(39)
Pig	Shock tube, live explosives in Humvee surrogate or building enclosure	Angiographic vasospasm immediately post-exposure in blast tube	Increased numbers of GFAP positive astrocytes	(40, 41)
Rat	Shock tube, 120 kPa	Transient increase in BBB permeability as judged by IgG immunostaining in superficial layers of cortex at 3 and 24 h post-exposure returning to normal 3 days after exposure	4-hydroxynonenal (4-HNE) and 3-nitrotyrosine (3-NT) increased at 3 h after exposure, returned to control levels at 24 h post-exposure, 5 and 10 days post-exposure increased microglial binding of ^3^H-PK11195 in contralateral and ipsilateral dentate gyrus and at 10 days in the contralateral ventral hippocampus and substantia nigra, microglial morphology characteristic of activated microglia in hippocampus and substantia nigra by CD11b/c immunostaining	(42)
Mouse	Shock tube, 13.9, 20.6, and 25 psi single blast overpressure (BOP1) or three repeated blast exposures (BOP3) at 20.6 psi with 1 and 30 min intervals between successive exposures	Frequent subdural hemorrhages with BOP3	Reactive oxygen species in cortex increased after BOP1 and BOP3	(37)
Rat	Shock tube, 20.6 psi whole-body exposure combined with 1 week stress (predator scent exposure combined with unpredictable stress)	2 months after blast/stress exposure, vascular endothelial cell growth factor (VEGF) elevated in hippocampus and prefrontal cortex	2 months after blast/stress exposure, increased GFAP and Iba-1 immunoreactivity in hippocampus and prefrontal cortex	(43)
Rat	Open-field exposure to 120 kg TNT at 48.9 kPa (7.1 psi, positive pressure, duration 14.5 ms) or 77.3 kPa (11.3 psi, 18.2 ms)	Narrowed vasculature in cerebral cortex at 1 and 4 days after blast	Iba-1 immunostaining for macrophages or microglia not different from control	(44)
Mouse	Open-field explosives, 4 and 7 m distance from blast (5.5 and 2.5 psi)	Increased BBB permeability 1 month post-blast on contrast enhanced T1-weighted MRI images at 5.5 psi but not 2.5 psi exposure, no change in BBB permeability 7 days post-exposure	Upregulation of manganese superoxide dismutase 2 in neurons and CXC-motif chemokine receptor 3 around blood vessels in fiber tracts at 1 month post-exposure	(45)
Mouse	Modular, multi-chamber shock tube capable of reproducing complex shock wave signatures, 68, 76, or 105 kPa exposures with durations 4–5 ms		Animals subjected to mild (68 kPa) or moderate (76 kPa) showed elevated mRNA for GFAP, myeloid related protein 8 (MCP-8), chemokine CC ligand-2 (CCL2/MCP1), and ED-1 in hippocampus and brainstem from 1–30 days post-exposure	(46)
Rat	Shock tube, 36.6 kPa (duration 4.1 ms), 74.5 kPa (4.8 ms) or 116.7 kPa (6.8 ms)	116.7 kPa overt threshold for pathology with 30% of rats having subdural hemorrhage and cortical contusions, all animals exposed to 116.7 kPa had pulmonary hemorrhages		(35)
Mouse	Shock tube, 77 kPa, positive pressure duration 4.8 ms	Microvasculopathy 2 weeks after exposure to blast with dysmorphic capillaries, thickened basal lamina, and swollen astrocytic end feet processes in the absence of macroscopic tissue damage or hemorrhage, perivascular tau accumulation	Activated microglia throughout brain, especially cerebellum 2 weeks after blast exposure based on *Ricinus communis* agglutinin immunostaining	(23)
Rat	Shock tube, 90–193 kPa, duration ~10 ms	Decreased cerebral blood flow in the internal cerebral vein by susceptibility-weighted imaging (SWI) 24 and 48 h at exposures of 117, 159, or 193 kPa or higher, reduced cerebral blood flow by continuous arterial spin-labeling (ASL) in hippocampus, auditory cortex, medial dorsal cortex, and thalamus		(47)
Rat	Shock tube, 230–380 kPa, subjected to on axis composite blast (blast wave plus pressure jet, duration 3–5 ms) or off axis (blast wave only, duration 50–100 μs) exposures		Gliosis in hippocampus as judged by GFAP immunostaining 1 and 7 days after both primary and composite blast	(48)
Rat	Shock tube, 120 kPa, positive pressure duration ~3 ms		Deposition of C3/C5b-9 in superficial layers of neocortex mostly around blood vessels at 3 and 48 h, elevated C3 by Western blot in neocortex at 3 and 48 h, CD45+leukocytes in neocortex at 3 and 48 h, increased TNF-α in neocortex at 3 h but not 48 h by Western blotting and immunostaining, increased aquaporin-4 expression in superficial layers of neocortex at 3 h, C3/C5b-9 deposition around neurons in the hippocampus	(49)
Monkey	120 kg TNT at 19 or 24 m (80 kPa or 200 kPa with ~10 ms duration)	By EM capillaries showed collapsed lumens, hypertrophic astrocyte end-feet, and vacuolated or electron dense endothelial cell cytoplasm, fluorescent perithelial cells (Mato cells) appeared to increase in number	Astrocytic hypertrophy	(50)
Rat	Shock tube, one or two 123 kPa exposures	6–24 h after exposure reduction of the BBB tight-junction proteins occludin, claudin-5, and zonula occludens 1 in brain microvessels with loss of the pericyte marker PDGF-β, activation of caspase-3 and cell apoptosis mostly around perivascular regions, increased permeability of Evans blue and sodium-fluorescein low molecular-weight tracers	6–24 h after exposure infiltration of immune cells across the BBB, induction of the free radical-generating enzymes NADPH oxidase 1 and inducible nitric oxide synthase along with evidence of oxidative and nitrosative damage (4-HNE/3-NT), activation of matrix metalloproteinases and fluid channel aquaporin-4	(51)
Rat	Shock tube, single or multiple (three) 74.5 kPa (duration 4.8 ms)	Frequent intraventricular hemorrhages after 24 h, no generalized histopathology in animals between 4 and 10 months after exposure but focal lesions resembling rips or tears found in many brains that frequently appeared to follow the lines of penetrating cortical vessels often disrupting cortical organization, microhemorrhages found within some but not most acute lesions	Microglial activation around focal cortical lesions	(52)
Mouse	20.6 psi, three with 1–30 min intervals between exposures	Histological evidence of constriction of blood vessels at 4 h after exposure	Altered RNA levels of multiple inflammation related genes including TNF family, interleukins and interleukin receptors, increased myeloperoxidase activity in cerebellum	(53, 54)
Rat	Shock tube, 129 kPa (duration 2.5 ms)		Increased reactive oxygen species in brain, upregulation of mRNA and protein expression of pro-inflammatory mediators, IFN-γ and MCP-1, microglial activation (increased Iba-1 immunostaining) at 2 weeks but not at 4, 24, or 48 h	(55)
Rat	Shock tube, single blast at 130, 190, 230, 250, and 290 kPa (impulses 184–452 Pa*s)	Diffuse BBB breakdown indicated by immunoglobulin G (IgG) immunostaining at 190 kPa and above sacrificed 24 h post-exposure		(56)
Rat	Shock tube, single or multiple (3) 74.5 kPa (duration 4.8 ms)	Microvascular pathology present at 24 h after injury within an otherwise normal brain parenchyma by electron microscopy, chronic changes in the microvasculature and altered collagen IV and laminin immunostaining of brain microvessels many months after blast exposure		(57)
Rat	Shock tube, single 120 kPa exposure		Elevated RANTES in cortex at 24 h after blast	(58)
Goat	Exposure to TNT in column-like buildings at 2–8 m from detonation, blast wave composed of two peaks from incident and reflected wave varying from 555/913 (peak/reflected) kPa at 2 m to 45/71 kPa at 8 m (durations 0.6–2.7 ms)	4 h after exposure at 2 m diffuse congestion of the vasculature on the surface of the brain with extensive subarachnoid and parenchymal hemorrhage visible grossly and microscopically, hemorrhagic component less pronounced but still present at 8 m exposures		(33)
Rat	Shock tube, 117 kPa, duration 7.5 ms		Increased number of GFAP-positive astrocytes in prefrontal cortex at 168 h after injury, no change in Iba-1-labeled microglia	(59)
Rat	Shock tube, 69, 97, and 165 kPa (10, 14, or 24 psi), positive pressure duration 2.5 ms		7 days post-injury activation of microglia in hippocampus of 69 kPa group, increased GFAP-positive astrocytes in hippocampus of 97 and 165 kPa groups	(60, 61)

**Table 2:** **Vascular and inflammatory effects on the nervous system following restricted cranial or thoracic exposure to blast in experimental animals**.

Species	Blast exposure	Vascular effects	Inflammatory effects	Other effects/effects of shielding	Reference
Rat	Shock tube, whole body blast (338.9 kPa, 52 ms duration) or local pulmonary blast (440 kPa, 50 ms duration)		Evidence of elevated oxidative stress and antioxidant enzyme defense systems (superoxide anion radical generation, increased malondialdehyde concentration, superoxide dismutase and glutathione activity) in hippocampus of animals in both groups at 3 and 24 h after exposure, mostly returning to normal by 5 days	Swollen neurons, glial reaction, and myelin debris in hippocampus by EM following whole body or local pulmonary blast, deficits in performance on an active avoidance task 3 h after injury, deficits in active avoidance task persisted at 5 days only in rats subjected to whole body blast	(62)
Pig	Local exposure to abdomen or top of skull, blast overpressure generated with air-compressed driven shock tube at levels of approximately 30 kPa in tube, and 14.4 kPa in air outside the abdomen, or 22 kPa in air outside the skull			Following abdominal exposure, maximal peak pressure in brain was 0.5 kPa in brain vs. 15 kPa in abdomen (brain 3% of that in the abdomen), 9 kPa in brain after direct skull exposure	(39)
Rat	Shock tube, 126 and 147 kPa exposures with or without Kevlar vest encasing thorax and part of the abdomen			Kevlar vest prevented widespread fiber degeneration that was prominent in brains of rats not protected by vest during a 126 kPa exposure	(63)
Rat	Shock tube, 358 kPa, duration 10 ms, head only exposure with body armor protection	Intracranial hematomas with brain swelling	GFAP accumulation in hippocampus 24 h after blast, still present at 30 days	Prominent silver staining in deep brain areas, increased GFAP and ubiquitin carboxy-terminal hydrolase L1 (UCH-L1) in CSF, body protection increased the threshold for mortality	(64)
Mouse	103 kPa (14.9 psi) whole body blast unprotected, or with chest and abdomen protected (body armor), or with head protected		Evidence of inflammation in brain as judged by bioluminescence imaging of myeloperoxidase (MPO) activity, MPO activation still present in brain 30 days after exposure without body protection	Head protection failed to prevent MPO activation in brain, body protection blocked blast-MPO activity in brain	(65)
Rat	Equivalent of 400 mg TNT detonated at various distances (100–400 kPa), exposed through portal in cabin that limited direct exposure to head	Acutely diffuse subarachnoid hemorrhage, contusions, and capillary damage in the cortex at 200 and 400 kPa exposures, minor/minimal injury at 100 kPa			(30)
Rat	Shock tube, 20.63 psi exposed as whole body blast with chest protection	Vascular endothelial cell growth factor (VEGF) increased in dorsal and ventral hippocampus at 71 days after exposure	IL-6 and IFN-γ levels elevated in amygdala and hippocampus but not prefrontal cortex 71 days after exposure, increased numbers of GFAP immunostained cells in the ventral hippocampus	Changes normalized by environmental enrichment	(66)
Rat	Shock tube utilizing compressed-air or helium, explosives oxyhydrogen, and cyclotrimethylenetrinitramine (RDX), 100–200 kPa, durations ~3–5 ms direct and ~6–7 ms reflected, rats fitted with Kevlar vest to protect the thorax with body protected by steel tube	Dilated blood vessels and hematomas visible grossly immediately after exposure			(31)
Mouse	Shock tube, 68 ± 8 kPa (9.9 ± 1.2 psi) static pressure, 103 kPa (14.9 psi) total pressure, with head or torso shielding			Without shielding multifocal axonal injury primarily in cerebellum/brain stem, corticospinal tract, and optic tract, prolonged behavioral and motor abnormalities including deficits in social recognition, spatial memory, and motor coordination, shielding of torso ameliorated axonal injury and partially protected against behavioral deficits, head protection not associated with any apparent benefits on the severity of axonal degeneration	(67)
Rat	Helium-driven shock tube, 35 psi (positive phase duration approximately 4 ms) applied to the left side of the head with body shielded	Increased BBB permeability suggested by IgG immunostaining at 24 h primarily affecting the contralateral cortex	GFAP, ED1, and Iba-1 immunostaining not prominently increased at 24 h, 72 h, or 2 weeks post-blast although small numbers of reactive microglia within areas of neuronal death	25% mortality due to impact apnea, surviving rats studied at 24 h, 72 h, or 2 weeks post-blast showed multifocal axonal degeneration by silver staining, deep cerebellar and brainstem white matter tracts most heavily affected, mild multifocal neuronal death at 24 and 72 h	(68)
Rat	Cranium only blast injury apparatus (COBIA) delivering blast overpressures generated by detonating 22 caliber cartridges of smokeless powder (450–700 kPa, complex pressure wave > 3 ms duration)	Widespread subarachnoid hemorrhages without cortical contusions or intracerebral or intraventricular hemorrhages, abnormal vascular immunolabeling for IgG in cerebellum, thalamus, and entorhinal cortex 1–7 days after blast exposure		Increased amyloid precursor protein, FluoroJade C and caspase-3 staining	(34)
Rat	Shock tube, 20.63 psi exposed as whole body blast with chest protection		51 days post-blast exposure elevated CRP, MCP-1, toll receptor 9, and GFAP in amygdala, prefrontal cortex, ventral and dorsal hippocampus, VEGF receptor fetal liver kinase 1 (FLK-1), claudin-5 and aquaporin-4 elevated in ventral hippocampus, FLK-1 and aquaporin-4 elevated in dorsal hippocampus and amygdala	Levels reduced by treatment with the non-steroidal anti-inflammatory drug minocycline for four consecutive days after blast exposure	(69)
Rat	Shock tube, one or five 138 kPa exposures with chest protection		GFAP immunostaining increased in hippocampus at 2 days post-exposure in single injured and at 22 days post-exposure in multiply injured		(70)
Pig	Shock tube, 24–37 or 40–52 psi in protective body armor	CSF VEGF elevated at 6 h, 72 h, and 2 weeks post-blast exposure but normal at 24 h, CSF von Willebrand factor increased at 6, 24, and 72 h but normal at 2 weeks			(71)
Ferrets	Shock tube, 98–837 kPa (durations from 2.1–14.1 ms) focused on head with thoracic and abdominal protection	Varying degrees of subdural, subarachnoid, and intracerebral hemorrhage, all worse at higher blast intensities		Apnea and death	(32)
Pig	Shock tube, blasts directed to unprotected head with lungs, and thorax protected using ballistic vests (110–740 kPa peak incident overpressure with durations from 1.3–6.9 ms)			Immediate apnea in 5 of 20 animals, no gross bleeding in brain, intracranial pressures ranged from 80–390 kPa which were lower than shock tube reflected pressures of 300–2830 kPa	(72)
Rat	Shock tube, head only, or chest only exposure (65, 110, 160, and 185 kPa)	Blood pressure in internal carotid artery rose 2–10 times physiological pressure of 14 kPa for ~2 ms after blast, rise correlated with level of blast	1 week post-exposure infiltration of CD68 + macrophages into brain	Pressure rise with chest only exposure 30% higher than head only, infiltration of CD68 + macrophages into the brain following chest exposure only	(73)
Rat	Tabletop shock tube, 31.47, 50.72, 72.05, and 90.1 psi (duration ~2 ms) delivered with thoracic and abdominal protection	Gross intracerebral hemorrhages 50.72 psi and above, immediate mortality with extensive intracranial bleeding at exposure to 90.1 psi and above	Increased number of GFAP expressing astrocytes and activated microglia in corpus callosum		(74)
Rat	Rifle primer-driven shock tube, primary blast at 145, 232, and 323 kPa (positive phase durations 14.2–55.8 μs), lungs protected by Kevlar vest	BBB disruption detected by IgG extravasation detected immunohistochemically with 232 and 323 kPa exposures at 24 and 48 h, small lesions scattered throughout brain, number and size of lesions correlated with peak overpressure level, despite laterally directed blast, equal numbers of lesions found in each hemisphere			(75)
Rat	Shock tube, 79 psi (1 ms duration) with or without shielding		Polymorphonuclear leukocytes (PMN) and lymphocytes infiltrated brain parenchyma within 1 h post-blast, GFAP, cyclo-oxygenase-2, interleukin-1β, and TNF-α present by 1 h and still detectable at 3 weeks post-injury, pro-inflammatory high mobility group box-1 protein detectable at 3 weeks	Greater 24 h infiltration of PMN and lymphocytes in non-shielded animals	(76)
Rat	Custom built blast simulator, 14 psi, repeated three times at 1.5 min intervals, body protected by a holding tube with head positioned perpendicular to nozzle of blast simulator		24 h after exposure increased 4-hydroxynonenal, (4-HNE) in the dorsal hippocampal commissure and forceps major corpus callosum, 7 days post-exposure increased GFAP expression in most brain regions	Antioxidant treatment (2,4-disulfonyl α-phenyl-tert- butyl nitrone and *N*-acetyl-cysteine) beginning 1 h after blast exposure reduced 4-HNE, and GFAP expression as well as upregulated amyloid precursor protein and neurofilament-68 expression	(77)
Rat	Live explosives (22 caliber cartridge of smoke-less powder), thorax-only blast injury apparatus (TOBIA, 451 kPa, duration 2 ms) or jugular-only blast injury apparatus (JOBIA, blast pressure applied to a jugular port, 59 mm Hg, ~400 ms duration pressure in jugular vein)	Immunolabeling 24 h after injury by TOBIA showed upregulation of TNF-α, ED-1, Sur1, and GFAP in veins or perivenular tissues and microvessels throughout brain	Upregulation of TNF-α, ED-1, Sur1, and GFAP	Blast injury induced by TOBIA caused apnea and diffuse bilateral hemorrhagic injury to lungs, perivenular effects by TOBIA prevented by ligating jugular vein and reproduced by JOBIA	(78)
Rabbit	TNT paper equivalent 600 mg, 638.2 kPa at 6.5 cm vertical distance from head, positive pressure duration 0.18 ms, body enclosed in wooden box with only head protruding	Frequent intracranial hemorrhages, increased BBB permeability based on Evans blue content in brain beginning at 6 h after injury, reaching peak at 48 h and remaining elevated at 3 days (last time point studied)	Elevated TNF-α and IL-8 peaking at 12–18 h	Transient apnea, bradycardia, and increased blood pressure immediately after exposure, treatment with hyperbaric oxygen reduced increases in BBB permeability and lowered levels of TNF-α and IL-8	(36)
Rat	Shock tube, five exposures administered as progressively higher exposures from 15.54–19.41 psi (107.14–133.83 kPa, durations 9.01–10.6 ms) at rate of 1 per 30 min with chest protection	2 days post-exposure elevation in one or more brain regions of VEGF and von Willebrand factor (vWF)	Two days post-exposure elevation in one or more brain regions of 4-HNE, hypoxia-inducible factor 1 α (HIF1α), aquaporin-4, integrin α6, Gal-1, MIP1, chemokine receptor 5 (CCR5), toll-like receptor 9, p38 mitogen-activated protein kinase and osteopontin, microglia and astrocyte markers (CD53/OX44), and GFAP increased	Transient depression of heart rate and oxygen saturation, 2 days post-exposure elevation of metallopeptidase inhibitor 1 (TIMP1), and matrix metalloproteinase 8 (MMP8)	(79)
Rat	Head-directed blast from a 27 caliber cartridge detonated 2 cm from head		Increased levels of IL-1β in hippocampus and thalamus and TNF-α in hippocampus at 6 h after injury, increased Iba-1 expressing microglia at 6 h and 30 days		(80)
Rat	Focal head exposure from bench-top blast wave generator (peak overpressure 885 kPa, rise time 4.7 ms)		8 h after exposure protein levels of erythropoietin, endothelial integrins, ICAM and sVCAM, and MCP-1 elevated in cortex, six inflammatory genes examined by qRT-PCR increased (complement C3, chemokines MCP5, MCP1, MCP3, I-PAC, and MDC)		(81)
Rat	Open-field electric detonator with 100 mg dinonyl-ortho- phthalate and 250 mg trimethylene-trinitroamine (equivalent of 400 mg TNT), rats fixed inside an aluminum shielded box with a 1 cm hole allowing frontal, parietal, and occipital parts of head exposed, detonator 7.5 cm from the center of the hole	Diffuse subarachnoid and intraparenchymal hemorrhage acutely	Brain nitric oxide (NO) and expression and activity of inducible nitric oxide synthase (iNOS) increased 2–24 h after exposure	Treatment with emodin, a possible inflammatory inhibitor, reduced pathology as well as NO and iNOS levels	(82)
Rat	Shock tube, blast wave directed to right side of skull with a polyvinyl chloride shield protecting rest of body, (peak incident overpressure of ~15 psi)	Increased BBB permeability at 6 h following blast as judged by Evans blue extravasation, increased VE-cadherin and occludin in brain, increased PKC isozymes in brain microvessels		Treatment with bryostatin-1, a PKC isozyme modulator decreased BBB breakdown	(83, 84)
Rat	Shock tube driven head-directed blast wave (66.5 to 94.0 psi, duration 10 ms)			Head directed exposure induced centrally mediated apnea acutely	(85)

While the hemorrhagic component is more apparent at high-level blast, pathologically hemorrhage also seems to be a feature of lower-level exposure. For example, Saljo et al. (39) observed small parenchymal and subarachnoid hemorrhages, predominately in the occipital lobe, cerebellum, and medulla oblongata in pigs at 3 and 7 days after relatively low-pressure exposures from a bazooka, Howitzer, or an automatic rifle in an open field. Gama Sosa et al. (52), studying rats at 24 h after single or multiple 74.5 kPa blast exposures while observing no generalized histopathology, observed frequent intraventricular hemorrhages.

Histologically, blast exposure can also induce a microvascular pathology in the absence of hemorrhage. Goldstein et al. (23), studying mice exposed to 77 kPa overpressures, observed a microvasculopathy 2 weeks after exposure with dysmorphic capillaries, thickened basal lamina, and swollen astrocytic end-feet in the absence of macroscopic tissue damage or hemorrhage. In rats exposed to single or multiple 74.5 kPa blast exposures, Gama Sosa et al. (57) observed a microvascular pathology 24 h after injury within an otherwise normal brain parenchyma. In these studies, chronic microvascular changes in the form of altered collagen IV and laminin immunostaining of brain microvessels was observed many months after blast exposure (57). Other studies have described vascular narrowing or constriction acutely after blast (44, 53).

Physiologically acute blast exposure causes prominent vasospasm (40) and decreased cerebral blood flow (47). Bauman et al. (40), studying pigs, demonstrated vasospasm angiographically immediately after exposure in a shock tube. Bir et al. (47), studying rats exposed to 90–193 kPa overpressures, found decreased cerebral blood flow in the internal cerebral vein by susceptibility-weighted imaging (SWI) at 24 and 48 h following exposures of 117 kPa or higher, as well as reduced cerebral blood flow by continuous arterial spin-labeling (ASL) in multiple brain regions for up to 72 h across the entire range of pressures.

At the functional level, acutely blast exposure has been associated with increased vascular permeability and blood-brain barrier (BBB) breakdown. Multiple studies have described at least transient increases in BBB permeability acutely as judged by IgG immunostaining (34, 42, 56, 68, 75). This staining, which has mostly been observed at higher blast exposures, typically returned to normal by 72 h, although Kuehn et al. (34) observed abnormal vascular immunolabeling for IgG in cerebellum, thalamus, and entorhinal cortex for as long as 7 days after blast exposure in rats. Several studies have documented increased BBB permeability to Evans blue (36, 51, 83, 84). Abdul-Muneer et al. (51) observed increased permeability of Evans blue and sodium-fluorescein low molecular-weight tracers 6–24 h after rats had been exposed to one or two 123 kPa exposures. Thus, a variety of studies have documented that functional breakdown of the BBB occurs acutely at least with relatively higher-level blast exposures. Whether chronic effects on BBB integrity exist has been less studied although one study (45) examining mice exposed to relatively low-level 5.5 psi overpressures generated by live explosives found increased BBB permeability 1 month post-blast on contrast enhanced T1-weighted MRI. Interestingly, these studies found no change in BBB permeability 7 days post-exposure, suggesting an evolving process that was not apparent acutely.

## The Pathophysiological Basis of Blast-Related Vascular Pathology

Morphological and functional data indicate that both large and small brain vessels are affected by blast injury. Little is known about the molecular changes associated with these abnormalities. Reductions of the BBB tight-junction proteins occludin, claudin-5, and zonula occludens 1 (ZO-1) in brain microvessels have been observed 6–24 h after exposure (51), although others have found increased occludin and VE-cadherin following blast (83, 84). Altered protein kinase C (PKC) isozymes have been observed in brain microvessels acutely following blast (84). Goldstein et al. (23) found perivascular accumulation of tau 2 weeks after blast exposure.

**Figure 1:**
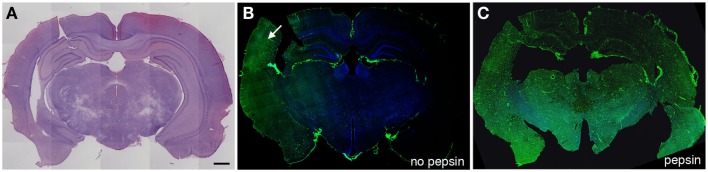
**Altered collagen IV immunostaining in the microvasculature of blast-exposed rats**. Shown are sections from a rat sacrificed 10 months after receiving three 74.5 kPa blast exposures delivered on consecutive days. (**A**) shows a coronal section stained with hematoxylin and eosin. (**B,C**) show adjacent sections immunostained with collagen IV without (**B**) or with pepsin (**C**) treatment. Immunostaining was performed as described in Gama Sosa et al. (57). Pepsin treatment (**C**) unmasks widespread collagen IV immunostaining. Arrow in (**B**) indicates a region that shows collagen IV immunostaining without pepsin pretreatment. Scale bar: 1 mm.

**Figure 2:**
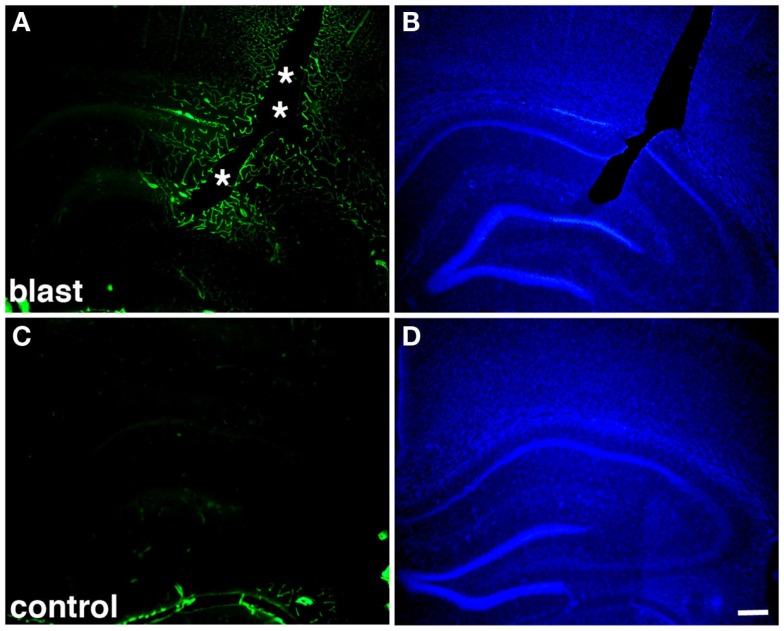
**Altered collagen IV immunostaining around blast-induced shear-related lesion**. Shown are sections from a rat sacrificed 10 months after receiving three 74.5 kPa blast exposures (**A–B**) or a non-blast exposed control (**C–D**). Sections were immunostained for collagen IV without pepsin pretreatment (**A,C**) and counterstained with 4’,6-diamidino-2-phenylindole (DAPI) (**B,D**) as described in Gama Sosa et al. (57). A focal blast-induced lesion (indicted by asterisks) is apparent in (**A,B**). Note the vascular staining with collagen IV in the blast-exposed animal despite the lack of pepsin treatment (**A**) in comparison to the unstained control (**C**). Scale bar: 250 μm.

**Figure 3:**
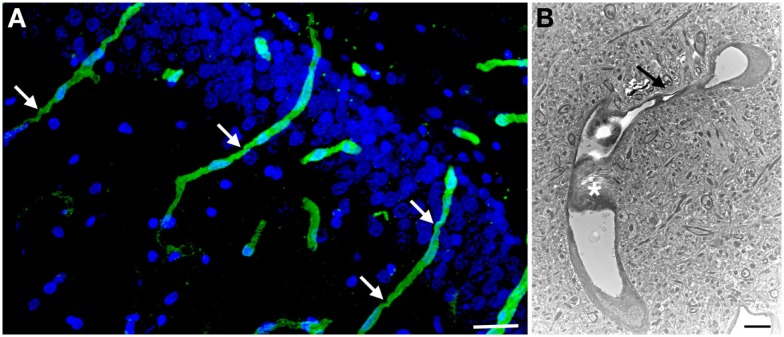
**Chronic microvascular pathology following blast exposure**. In (**A**) a section of the hippocampal dentate gyrus is shown from a rat sacrificed 6 months after receiving three 74.5 kPa blast exposures. Sections were immunostained for collagen IV without pepsin pretreatment and counterstained with DAPI as in Figure 2. Note the prominent vascular staining despite the lack of pepsin treatment. Arrows indicate strictures in the vessels. In (**B**) an electron micrograph is shown taken from the frontal cortex of a rat that received three 74.5 kPa blast exposures and was sacrificed 6 months after the last exposure. Note the amorphous material in the lumen creating a near complete occlusion (asterisk). The vessel also becomes narrowed (arrow). The brain parenchyma surrounding the vessel appears normal. Electron microscopy was performed as described in Gama Sosa et al. (57). Scale bar: 25 μm (**A**); 2 μm (**B**).

The long-term effects of blast on the vasculature are likewise poorly understood, although one study observed altered immunostaining of the microvasculature with collagen IV and laminin several months after blast exposure (57) (Figures 1–3). This immunostaining, which occurred without the partial proteolytic digestion needed to unmask such antigens in perfusion-fixed tissue of adult rat brain (86), suggests a loss of the normal tightness of the gliovascular junctions and ongoing vascular remodeling (57). Why vascular remodeling would continue many months after blast exposure is unknown but suggests a chronic pathology. Beyond these findings, the molecular basis for blast-related vascular pathology remains poorly understood. Further examination of the molecular changes occurring particularly in the chronic phase is needed to understand the pathophysiological basis of blast-related vascular injury.

As noted above, acute blast exposure is associated with prominent vasospasm (40). Vasospasm would be expected to lead to decreased blood flow, which has been documented acutely *in vivo* (47). Sato et al. (87) have also suggested that a blast wave applied directly to the brain can induce acute physiological changes. In these studies, a laser-induced shock wave was applied directly to the rat brain through the skull under conditions where no changes in systemic physiological parameters occurred. Blood vessels at the brain surface showed vasodilatation for 3–4 min followed by long-lasting vasoconstriction. These changes were associated with electroencephalographic evidence of spreading depression, long-lasting hypoxemia, and signal changes indicating mitochondrial energy impairment.

In considering potential transcranial effects of blast, focused ultrasound (FUS) serves as an interesting comparison. Similar to blast, FUS involves generation of a compression-rarefaction longitudinal wave. However, in contrast to blast, a FUS longitudinal wave has a higher frequency content than a blast wave, and is applied focally in a typically pulsed fashion leading to generally higher peak pressures than a blast wave (56, 88). Yet, similar to blast, Raymond et al. (89) demonstrated BBB disruption and acute vasospasm following direct transcranial application of 10 ms bursts of ultrasound at 1 MHz. Hynynen et al. (88) similarly showed BBB disruption following direct transcranial application of 10 ms bursts of ultrasound at 260 kHz. Sheikov et al. (90) demonstrated loss of tight junction proteins occludin, claudin-5, and ZO-1 by immunoelectron microscopy following application of 10 ms bursts of ultrasound at 1.5 MHz. Thus despite differences in the physical characteristics of the two exposures, the biological effects have interesting similarities.

Alford et al. (91, 92) have suggested that blast-induced vasospasm may play another role by initiating a phenotypic switch in vascular smooth muscle cells that has long-term consequences. They employed high-velocity stretching of engineered arterial lamellae to simulate the mechanical forces of a blast pulse on the vasculature. One hour after a simulated blast, injured tissue displayed altered intracellular calcium dynamics leading to hypersensitivity to a contractile stimulus with endothelin-1. One day after simulated blast, tissues exhibited a prolonged hypercontraction and changes in vascular smooth muscle cells suggestive of a phenotypic switch that was blast force-dependent. They argued that blast-induced cerebral vasospasm causes a mechanically transduced genetic switch in vascular smooth muscle cells that potentiates vascular remodeling leading to further vasospasm and lumen occlusion. Although this phenomenon has yet to be demonstrated *in vivo*, blast effects on large cerebral blood vessels acutely are well established (40). Another mechanism would need to be invoked to explain effects on the microvasculature where a smooth muscle layer is lacking.

## Thoracic and Systemic Effects of Blast on the Nervous System

The primary blast wave may affect the brain as it is transmitted through the tissue. Blast waves also cause acceleration/rotation of the head imparting mechanical energy to the brain that may cause injuries similar to those seen in non-blast TBI. A third mechanism that has been considered is that the blast wave striking the body may cause indirect CNS injury through what has been referred to as a thoracic or systemic mechanism (65, 93). Support for this mechanism came initially from observations in animals suggesting that peripheral injuries by conventional ballistics could cause indirect CNS damage through pressure waves transmitted from the peripheral impact site to the CNS. Studies in pigs, for example, showed that high-frequency pressure waves could be recorded in the brain of an anesthetized pig that was shot in the thigh with a projectile (94–98). Along with pressure changes, microscopic damage was detected in the hippocampus and cerebellum (97). Studies in dogs produced similar results (99). In rats exposed to 12 low-level blast exposures (34.5 kPa), we (unpublished data) have noted a stripping away of the intra-vascular glycocalyx, evidence consistent with increased hydrostatic pressure resulting from the blast event.

Cernak and collaborators first brought attention to a possible systemic mechanism in blast (62, 65, 100). In rats subjected to a local pulmonary blast, they found electron microscopic evidence of swollen neurons, a glial reaction, and myelin debris in the hippocampus following either whole body or local pulmonary exposure (62). With both exposures, deficits were noted in an active avoidance task along with evidence of oxidative stress and activation of antioxidant enzyme defense systems in hippocampus immediately following the insult. Initially, thoracic effects were thought to be vagally mediated, as bilateral transections of the vagus, glossopharyngeal, and hypoglossal nerves in rabbits were found to prevent the apnea, hypotension and bradycardia, as well as mitigate some of the metabolic changes in brain (100). Irwin et al. (101) observed similar effects of vagotomy in rats.

More recently, the mechanism of the thoracic/systemic effect has been modified to emphasize blast wave transmission through the vasculature (65, 102, 103). Specifically, it was proposed that a high-pressure blast wave hitting the body compresses the abdomen and chest inducing oscillating high-pressure waves that can be transmitted through the systemic circulation to the brain. In brain, preferential damage to cellular elements close to cerebral vessels was suggested to damage the cerebral vasculature and disrupt the BBB. The key element of this proposal is that the kinetic energy of the shock wave is transferred into hydraulic energy that is carried through the cardiovascular system causing rapid blood displacement into the lower pressure intracranial compartment. Clearly, if correct, this injury mechanism has implications not only for understanding blast-related pathophysiology but also for design of protective body armor.

Subsequently, a number of studies have addressed the relative importance of body vs. head exposure using selective shielding or a variety of designs for selective exposure. These studies are summarized in Table 2. Among them, multiple studies support the notion that shielding the body can block some blast effects on the CNS (63, 65, 67, 78). For example, Long et al. (63) showed that fitting rats with a Kevlar vest encasing the thorax and part of the abdomen reduced mortality as well as prevented widespread axonal degeneration seen in brains of unprotected rats exposed to a whole body blast. Koliatsos et al. (67), studying mice exposed to a whole body blast, found that without shielding there was multifocal axonal injury as well as deficits in social recognition, spatial memory, and motor coordination. Shielding of the torso reduced axonal injury and partially protected against behavioral deficits, while head protection was not associated with any apparent benefits on the severity of axonal degeneration (67). Studies in mice have found that head protection fails to prevent an inflammatory response in brain as judged by bioluminescence imaging of myeloperoxidase (MPO) activity, while body protection blocked blast-induced MPO activity in brain (65).

Simard et al. (78) have provided support for the systemic effect occurring through a vascular mechanism. These authors constructed devices that allow selective blast exposures to the thorax or the jugular veins of rats. A thorax-only device delivers a collimated blast wave to the right lateral thorax of the rat, precluding direct impact on the cranium. The jugular device delivers a blast wave to the fluid-filled port of an extracorporeal intravenous infusion device whose catheter is inserted retrogradely into the jugular vein, precluding lung injury. Thorax-only exposure caused apnea with diffuse and bilateral pulmonary hemorrhage. At 24 h after thoracic exposure, immunolabeling found that around veins, perivenular tissues, and microvessels in brain there was upregulation of tumor necrosis factor-α (TNF-α), ED-1, sulfonylurea receptor 1 (Sur1), and glial fibrillary acidic protein (GFAP). Perivenular inflammatory effects induced by thorax-only exposure were prevented by ligating the jugular vein and were reproduced by the jugular exposure, all consistent with blast injury to the thorax leading to perivenular inflammation and a reactive gliosis on the basis of a hydrodynamic pulse transmitted through the vasculature (78). Consistent with the systemic effect being cardiovascular, studies in rats have shown that blood pressure in the internal carotid arteries rises 30% more following a selective chest exposure than a brain exposure (73).

In contrast, other studies clearly document that head exposure alone without body exposure induces significant injury to the nervous system (30–32, 34, 36, 63, 64, 66, 68–72, 74, 75, 79–85, 104–106). Indeed, it seems that use of some form of body shielding or selective head exposure has become the norm for blast exposure experiments in many laboratories. Examples include that cranium-only blast exposure in rats leads to widespread subarachnoid hemorrhage with abnormal vascular immunolabeling for IgG, increased amyloid precursor protein staining, and scattered cell death (34) (Table 2). Garmen et al. (68) found that following a blast applied to the left side of the head with the body shielded, 25% of rats died due to impact apnea. Surviving rats studied at 24 h to 2 weeks post-blast showed multifocal axonal degeneration along with scattered neuronal death and increased BBB permeability that primarily affected the contralateral cortex. Studies in rats and ferrets across a range of pressures found that exposures focused on the head with thoracic and abdominal protection caused varying degrees of subdural, subarachnoid, and intracerebral hemorrhage, as well as apnea and death, especially at higher level exposures (32, 74). Pigs and rats experience frequent apnea after blast exposure to the unprotected head (72, 85). Head-directed blast may also induce systemic effects as Prima et al. (105) found that in rats fitted with body armor protection, thrombin generation in blood increased along with changes in other peripheral markers.

Therefore multiple studies show that body shielding can reduce or eliminate the adverse effects of blast on the brain (63, 65, 67, 78) with the studies of Simard et al. (78), perhaps providing the most direct evidence for transmission of the pressure wave through the vasculature. Thus, support exists for a thoracic mechanism playing a substantial role at least under some experimental conditions. In contrast, multiple other studies document that blast exposure of the head alone can reproduce a range of CNS pathology (30–32, 34, 36, 63, 64, 66, 68–72, 74, 75, 79–85, 104–106).

Unfortunately, many technical factors related to the characteristics of the blast wave produced by different shock tubes as well as variations in specimen mounting and degree of head restraint among other factors limit comparisons between studies even within the same species (12). Yet, in looking for differences that may explain the seemingly divergent findings summarized in Table 2, it is noteworthy that the studies using head exposure alone have typically reported adverse effects at higher blast pressures than those used in studies involving whole body or systemic exposures. For example, Turner et al. (74) subjected rats to 31.47, 50.72, 72.05, and 90.1 psi exposures, (duration ~2 ms, ~216.98–621.22 kPa) delivered with thoracic and abdominal protection using a tabletop shock tube. They found gross intracerebral hemorrhages with the 50.72 psi exposure and above but none at the 31.47 psi exposure, a pressure within a range that commonly leads to gross cerebral hemorrhages with whole body exposures (35). It is therefore tempting to speculate that a threshold may exist above which isolated cranial exposures are sufficient to cause significant CNS injury but below which the effects of systemic/thoracic mechanisms are critical. Whether thoracic effects play a role in the low-level blast range including mTBI and subclinical blast is unclear. Answering this question in part depends on how mTBI is defined in animal models, a subject that has recently been discussed elsewhere (12). It depends as well on assessing outcomes more subtle than intracerebral hemorrhage.

Whether all vascular pathology associated with low-level blast exposure in animal models can be explained by a thoracic/systemic mechanism is also unclear. Relatively low-level blast pressure waves (~35 kPa) are transmitted to brain even when the body is protected (107). Other studies have found that rats exposed to whole body 74.5 kPa exposures, while developing no general histopathology, exhibited focal cortical lesions that likely represent shear-related effects due to pressure differentials transmitted through the perivascular spaces in the brain (52). This conclusion was drawn based on observations that the focal lesions typically followed the course of penetrating cortical vessels but were seldom associated with hemorrhage as would be expected if the pressure wave was transmitted through the vasculature. In contrast, hemorrhages might not occur if the blast pressure wave was transmitted through the vascular compartment but not through the blood vessel lumen, an effect that could occur if the main pressure wave was transmitted through the Virchow–Robin compartments. Many studies have documented increases in intracranial pressure acutely following blast exposure (23, 39, 40, 72, 107–114). Increased CSF pressure transmitted through the Virchow–Robin compartments could generate local pressure differentials at the interface between the vascular basal lamina and the surrounding tissues. Shearing along this plane would conceptually leave the blood vessel wall intact preventing hemorrhages. Disruption of the Virchow–Robin compartments could have another effect in that recently a brain-wide network of paravascular channels has been identified, termed the “glymphatic” pathway, along which CSF moves through the brain parenchyma (115). This pathway which facilitates clearance of interstitial solutes (115) would likely be disrupted by blast driven pressures transmitted through the Virchow–Robin compartments.

## Inflammation in Blast-Induced Brain Injury

Inflammatory changes in brain after blast exposure in animals are well documented (Tables 1 and 2). Blast exposure induces a number of cytokines/inflammatory mediators including interleukin-6 (IL-6), interleukin-8 (IL-8), interleukin-1β (IL-1β), interferon-γ (IFN- γ), C-reactive protein (CRP), monocyte chemotactic protein 1 (MCP-1), macrophage inflammatory protein 1 (MIP1), galectin-1 (Gal-1), TNF-α, and toll receptor 9 (36, 49, 66, 69, 76, 78–80). cDNA microarray studies find altered RNA levels of multiple inflammation related genes including TNF family-related genes, interleukins, and interleukin receptors (54).

At the cellular level, microglial activation has been reported in many studies (23, 38, 42, 52, 55, 60, 61, 74, 79). Increased numbers of GFAP-positive astrocytes (48, 59–61, 66, 70, 74, 76, 77) or elevated GFAP levels in brain (41, 64, 69, 78, 79) have also been reported. Polymorphonuclear leukocytes and lymphocytes can infiltrate brain parenchyma within 1 h of a blast exposure (76) and CD45+ leukocytes have been found increased in cortex 3 and 48 h after blast (49). Elevated cortical C3 levels and perivascular deposition of complement C3/C5b-9 has been observed in superficial cortical layers (49). Evidence of oxidative stress which is commonly found in association with inflammation is also seen following blast (37, 42, 51, 55, 62, 77, 79, 82).

Inflammatory changes in brain have been studied mostly following acute blast exposure with some of the changes being short-lived. For example, TNF-α while increased in cortex at 3 h was not increased at 48 h (49). However, Kovesdi et al. (66) found that IL-6 and IFN-γ were elevated in amygdala and hippocampus for at least 71 days after blast exposure and another study (69) found elevated toll receptor 9, MCP-1, and CRP, 51 days post-exposure, suggesting that some changes persist. At 1 month after exposure, Rubovitch et al. (45) also observed increased perivascular expression of the CXC-motif chemokine receptor 3 which regulates leukocyte trafficking across the vasculature. Consistent with chronic cellular infiltration in brain, MPO bioluminescence imaging suggests polymorphonuclear leukocyte accumulation for at least 30 days after blast exposure (65).

In animal models, blast is also associated with markers of inflammation in blood and plasma (Table 3). Elevations in CRP, IL-1 and IL-10, plasma complement levels, blood and plasma MPO, and TNF-α have all been observed (48, 49, 53, 58, 69, 79, 106). Kamnaksh et al. (79) recently identified a range of elevated inflammatory, extracellular matrix, vascular and oxidative stress related proteins in plasma after blast exposure. As in brain, many of the changes have been short lived. For example in one study, elevations of IL-1β, TNF-α, and IL-10 while detectible at 3 h post-exposure peaked at 24 h and returned to normal by 48 h (58), although as in brain, elevations in CRP and MCP-1 have been reported in blood 51 days after blast exposure (69).

**Table 3:** **Changes in blood or plasma in experimental animals after blast**.

Species	Blast exposure	Findings	Reference
Rat	Shock tube (358 kPa, duration 10 ms), head only exposure with body armor protection	GFAP, neuron specific enolase (NSE), and UCH-L1 increased in blood	(64)
Pig	Shock tube, 20–40 psi	Increased serum levels of S100B, MBP, NSE, and NF-H 6 h to 2 weeks following injury	(116)
Rat	Shock tube, 20.6 psi whole body exposure combined with 1 week stress (predator scent exposure combined with unpredictable stress)	2 months after blast/stress exposure elevated serum levels of cortisol, creatine kinase-BB, neurofilament-H (NF-H), neuron specific enolase (NSE), glial fibrillary acidic protein (GFAP), and vascular endothelial cell growth factor (VEGF).	(43)
Rat	Shock tube, 20.63 psi	51 days post-blast exposure, elevated CRP, MCP-1, cortisol, NSE, neurofilament-H, tau, claudin 5, and S100β in serum, elevations normalized by daily treatment with non-steroidal anti-inflammatory drug minocycline for four consecutive days after blast exposure	(69)
Rat	Shock tube, 230–380 kPa on axis composite blast (blast wave plus pressure jet, duration 3–5 ms) or off axis (blast wave only, duration 50–100 μs) exposures	Increased serum GFAP 1–7 days after primary and composite blast, markers of vascular/endothelial inflammation integrin α/β, soluble intercellular adhesion molecule-1, and L-selectin increased in serum within 6 h after primary and composite blast persisting for 7 days, systemic IL-1, IL-10, and fractalkine raised predominantly after primary blast exposure	(48)
Rat	Shock tube, one or five 138 kPa exposures	Serum VEGF, NSE, neurofilament H, and GFAP elevated in single and multiply injured animals at 22 days post-exposure	(70)
Rat	Shock tube, 117 kPa, duration 7.5 ms	Decreased IL-1a at 3 h, decreased macrophage colony stimulating factor (m-CSF) at 24 h, increased EPO at 48 h, decreased IL-1a, IL-1ss, IL-6, IL-10, EPO, and increased VEGF and m-CSF at 72 h, no changes in TNF-α at any time point	(117)
Rat	Shock tube 120 kPa, positive pressure duration ~3 ms	Plasma C5b-9 elevated by ELISA at 3 h and 24 h after blast but not 72 to 168 h	(49)
Rat	Shock tube, 138 kPa, single or repeated (5 total administered on consecutive days)	Changes in arterial oxygen saturation levels and heart rates of single-injured and multiply injured rats throughout observation period of 42 days, elevation of plasma biomarkers at 42 days (HNE, HIF-1α, ceruloplasmin, VEGF, von Willebrand factor, neurofilament H, GFAP, myelin basic protein, MMP-8, formyl peptide receptor 1, p38 mitogen-activated protein kinase, and chemokine receptor 5) in one or more groups	(118)
Mouse	Shock tube, single 10, 15, or 21 psi exposures, repeat 3 × 21 psi delivered with 1–30 min intervals between exposures, whole body or head restricted exposure with a vest covering whole body except the head	Plasma aspartate aminotransferase, alanine aminotransferase, lactate dehydrogenase, and creatine kinase increased as early as 1 h after blast exposure remaining elevated up to 6 h in an overpressure dose-dependent manner, returning close to normal levels at 24 h, head-only blast exposure with body protection showed no increase in the enzyme activities.	(104)
Mouse	Shock tube, 20.6 psi three times with 1–30 min intervals between exposures	Increased platelet activation at 4 h after repeated blast exposures, platelet serotonin decreased at 4 h after blast with a concurrent increase in plasma serotonin levels, blood and plasma myeloperoxidase enzyme activity and expression increased in repeated blast exposed mice at multiple time-points	(53)
Rat	Head-directed blast with body armor delivered as (1) moderate “composite” blast with strong head acceleration or (2) moderate primary blast, without head acceleration, 230–380 kPa	Thrombin generation in blood increased in both forms of blast, integrin alpha/beta and sICAM-1 levels elevated after both composite and primary blast at 6 h, 1 d, and 7 d, sE-selectin exhibited near normal levels after composite blast but increased at 7 d after primary blast, MMP-2, MMP-8, and MMP-13 rose slightly after composite blast and increased two-to-fourfold after primary blast	(105)
Rat	Shock tube, single 120 kPa whole body exposure	Increased IL-1β, erythropoietin, TNF-α, and IL-10 in the serum at 3 h, reaching peak at 24 h and returning to normal at 48 h	(58)
Rat	Shock tube, five exposures administered as progressively higher exposures from 15.54–19.41 psi (107.14–133.83 kPa, durations 9.01–10.6 ms) at rate of one per 30 min with chest protection	Two days post-exposure elevation in plasma of 4-HNE, HIF-1α, ceruloplasmin, VEGF, von Willebrand factor, aquaporin 1 and 4, fetal liver kinase 1 (FLK1/VEGF receptor 2), claudin 5, integrin α 6, TIMP1, TIMP4, Gal-1, p38 mitogen-activated protein kinase, MIP1, chemokine receptor 5, MCP1, cytokine-induced neutrophil chemoattractant 1 (CINC1), fibrinogen, CRP, *N*-formyl peptide receptor, GFAP, OX44, S100β, neuron-specific enolase, neurofilament H, creatine kinase-brain type, and tau	(79)
Rat	Exposure to pentaerythritol tetranitrate explosive, (2,4,6-trinitrotoluene equivalent = 15.6 mg, “moderate” blast or = 27.0 mg “severe” blast) using a spherical exploder fixed over the head	After either moderate or severe injury serum levels of tau, GFAP and TNF-α increased at 8 h, reaching peak at 24 h and remaining elevated at six days (last time-point tested), serum malondialdehyde levels increased at 3 and 6 days	(106)

## Linking Vascular and Inflammatory Factors to the Neuropsychiatric Features of Blast-Related TBI

One of the striking features of the mTBI cases in veterans returning from Iraq and Afghanistan is the frequent presence of PTSD. PTSD or depression is present in over one-third of Iraq veterans who suffered mTBI (8, 119). The presence of PTSD has complicated diagnosis as the clinical distinction between a post-concussion syndrome and PTSD is often difficult (3). While the frequent overlap could represent dual exposures to TBI as well as PSTD-related psychological stressors, other studies have suggested that many of the adverse physical or neuropsychological outcomes following blast-related mTBI are only non-specifically related to blast or better explained by PTSD (12). However, several studies now show that blast exposure in animals can induce PTSD-related traits in the absence of a psychological stressor (120–122). Blast exposure in animals has been found to induce anxiety, increased acoustic startle, increased prepulse inhibition, altered fear conditioning, and enhanced responses to a predator scent challenge (12, 120–122), with one study finding that these traits were still present many months after blast exposure (120). Human neuroimaging studies also show that blast-related mTBI is associated with chronic effects that are unlikely to be explained by comorbid PTSD (12). Thus, multiple lines of evidence support the notion that blast exposure is associated with chronic neurobehavioral effects independent of PTSD.

Whether vascular and inflammatory factors play a role in neurobehavioral effects associated with blast injury is unknown. Supporting a potential role is a substantial literature suggesting that chronic low-grade inflammation is a consistent feature of many neuropsychiatric disorders including major depression and PTSD (123, 124). Epidemiological studies suggest inflammation as a risk factor for major depression (123). Multiple studies have reported increased circulating lymphocytes and phagocytic cells in major depression along with elevated plasma levels of acute phase reactants such as CRP and increased proinflammatory cytokines, including interleukins IL-1β, IL-2, and IL-6, and TNF-α (123). Levels of oxidative stress are also linked to inflammation and major depression (125). Relatively similar changes occur in PTSD where greater total lymphocytes, increased number and activity of natural killer (NK) cells, and activated T lymphocytes have been noted along with increased plasma levels of CRP and proinflammatory cytokines including IL-1β, IL-6, and TNF-α (124, 126). Inflammatory changes may in addition be linked to alterations in the hypothalamic pituitary axis (HPA) found in PTSD and recently epigenetic changes in genes related to the HPA axis have been found that may affect peripheral immune reactions (124).

How inflammation is related to the clinical features of major depression and PTSD is not clear. Some of the most compelling evidence for cytokine production playing an etiological role comes from patients treated with interferons which have documented high rates of depression with either IFN-α or IFN-γ (127). Proinflammatory cytokines also influence pathways affected in major depression including serotonin, noradrenalin, and kynurenine metabolism, all of which have links to neurotransmitter systems implicated in depression (123).

**Figure 4:**
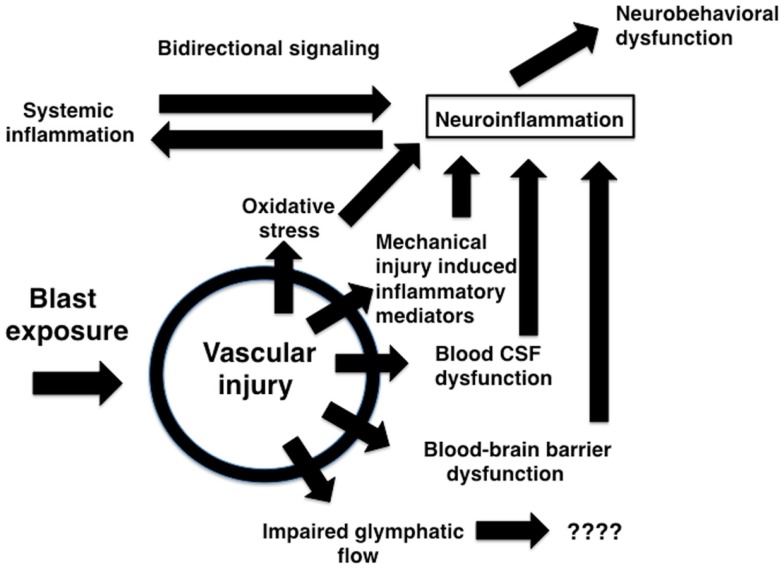
**Potential mechanisms relating blast-induced vascular injury to neuroinflammation and neurobehavioral dysfunction**. Mechanical injury to blood vessels induces local production of inflammatory mediators including cytokines, chemokines, and cell adhesion molecules through largely cell-mediated mechanisms. Mechanical injury also induces oxidative stress which can be associated with induction of an inflammatory response. Changes in BBB permeability could support initiation of an inflammatory reaction acutely and help sustain a response chronically. Blast-induced damage to the choroid plexus may alter blood-CSF barrier function which has been linked to induction of an inflammatory response. Impaired BBB function and bidirectional signaling between CNS and systemic inflammatory responses could amplify both reactions. Chronic immune activation could lead to neurobehavioral changes in the absence of direct neuronal pathology. Vascular pathology could also disrupt recently described glymphatic pathways that move CSF through the brain parenchyma.

The brain vasculature is a central target of blast-induced effects (Tables 1 and 2) with recent studies suggesting that the cerebral vasculature is selectively vulnerable (57). Blast exposure is associated with chronic inflammatory changes in brain and blood in experimental animals (Tables 1–3) with chronic changes in the microvasculature being apparent many months after blast exposure (57) (Figure 3), at a time when persistent behavioral traits are present (120). Figure 4 summarizes how an initial vascular insult might lead to chronic neurobehavioral effects. Mechanical injury to blood vessels might induce local production of inflammatory mediators including cytokines, chemokines, and cell adhesion molecules, effects that could occur through cell mediated mechanisms activated by physical trauma (128). Oxidative stress induced by mechanical injury and other factors is also associated with induction of an inflammatory response (125). Changes in BBB permeability could have various functional consequences including potentiating an inflammatory reaction. Blast also damages the choroid plexus (52, 129), which has been associated with inflammatory responses due to altered blood-CSF barrier function (130).

Chronic vascular pathology could have another effect in that recently a brain-wide network of paravascular channels has been identified, termed the “glymphatic” pathway along which CSF moves through the brain parenchyma (115). Through this pathway substances including the amyloid β and tau proteins are transported out of the CNS (115). Interestingly transport through this pathway is impaired in mice with a null mutation of the astroglial water channel aquaporin-4 and multiple studies have identified changes in aquaporin-4 expression following blast injury (49–51, 131). Multiple studies have also identified accumulation of tau (23, 58, 66, 132, 133) following blast exposure although β-amyloid levels are decreased acutely following blast injury (134). In non-blast TBI models, it has recently been shown that one route, whereby serum markers such as S100β, GFAP, and neuron-specific enolase reach the systemic circulation is through the brain glymphatics and the cervical lymph nodes (135).

The cellular response following blast includes a microglial and astroglial reaction. Microglia play central roles in brain inflammatory responses and could therefore play critical roles in activating a brain inflammatory response following blast (136, 137). Astrocytes also regulate BBB permeability and influence microglial activation (123). Chemokines released by astrocytes recruit monocytes and macrophages as well as other immune cells into the CNS (123), and astrocytes are activated by TNF-α, IFN-γ, IL-1, and IL-6 (123). Activated microglia produce proinflammatory cytokines including IL-1β, IL-6, and TNF-α (138, 139). A microglial response often precedes the activation of astrocytes and is thought to be involved in maintaining an astroglial response (140). Inflammatory signaling through microglia has been linked to serotonin metabolism through effects on the kynurenine pathway leading to altered 5-hydroxytryptophan availability and lower levels of serotonin (127). Microglial activation is also a feature of non-blast TBI (141).

An additional cellular element that likely plays a role in this process is the pericyte, which is damaged acutely in blast-associated vascular injury (57). Pericytes have a close structural relationship with endothelial cells and regulate capillary permeability through mechanical means, as well as secretion of cytokines and production of nitric oxide and matrix metalloproteinases (142). Pericytes are increasingly being recognized as having a role in normal physiology as well as disease (143). Like astrocytes, pericytes regulate the BBB and control leukocyte migration into brain through production of soluble factors as well as effects on adhesion molecule expression (142). Under physiological conditions, pericytes appear to function as immune-suppressors, supporting the notion that their loss or damage secondary to blast increases immune activation in brain.

Markers of both a central and a peripheral inflammatory response are found after blast injury (48). While the relationships between the central and peripheral inflammatory responses after blast exposure are not known, one possibility is that the systemic inflammatory response serves at least in part as a driver of the CNS response, an effect that could be accentuated in the presence of an impaired CNS vasculature following blast exposure. Cytokines cross the BBB either through active transport or damaged endothelium (144). Thus the CNS can be affected not only by inflammatory mediators produced within the brain, but as well through actions of mediators originating from the periphery. Interestingly, increased BBB permeability along with elevated intrathecal production of IgG is found in a subgroup of patients with major depression (123). These changes seem most prominent in patients with the most treatment-resistant symptoms, supporting the notion that a peripheral inflammatory response might drive a chronic CNS inflammatory response. Chronic immune activation in the brain following vascular injury could lead to neurobehavioral changes in the absence of direct neuronal pathology.

Thus, evidence collectively points to the need to develop strategies to prevent or repair vascular damage following blast injury. It also points to targeting the immune response. Inflammation is a recognized component of non-blast TBI (145, 146) and reversal of established anxiety-like behavior in rats after a lateral fluid percussion injury has been reported following immunomodulatory therapy (147). While studies following blast exposure are limited, one has reported that treatment with minocycline, a drug whose effects include anti-inflammatory properties, can reverse behavioral deficits and neurochemical changes in a rat model of blast-related TBI (69). Another study found that a complement inhibitor could attenuate many of the effects of acute blast injury including BBB breakdown (58), while Du et al. (77) found that treatment with a combination of antioxidants could reduce blast-induced brain injury. In addition, microglial activation is becoming increasingly recognized as a factor in many neurological diseases and might be another therapeutic target (136, 138, 139).

## Conclusion

The possible effects of blast exposure on the nervous system have been discussed since WWI. Public interest in this topic as well as interest in the scientific community has expanded rapidly in recent years because of the prominence of blast-related TBI in the conflicts in Iraq and Afghanistan. In humans, high-pressure blast exposure can cause extensive CNS injury with a prominent hemorrhagic component. Less is known about the pathology of human blast-related mTBI, and animal studies are the source of most knowledge about low-level blast exposure. As in humans, acute high-level blast exposure in experimental animals is associated with a prominent hemorrhagic component. At the functional level, high-level blast exposure acutely disrupts the BBB. Low levels of blast exposure are also associated with a microvascular pathology that can be seen in the presence of an otherwise normal brain parenchyma, suggesting that the vasculature may be selectively vulnerable to blast injury. Multiple studies have shown that shielding the body can reduce or eliminate the adverse effects of blast on the brain supporting the role for thoracically mediated effects whereby pressure waves transmitted through the systemic circulation cause damage in brain.

Inflammatory changes after blast exposure in animals are also well known. In brain, blast exposure induces a number of inflammatory mediators including many interleukins, interferons and cytokines. At the cellular level, microglial and astroglial reactions occur. In animal models, blast is associated with increases in inflammatory markers in blood and plasma including many of the factors elevated in brain. Evidence of oxidative stress which is commonly found in association with inflammation has also been documented in brain and blood.

Thus much evidence supports a role for vascular and inflammatory factors in the pathophysiology of blast-related brain injury, although most animal studies have utilized relatively high-level blast exposures that likely more approximate human moderate to severe TBI than mTBI. Where information is limited is on the effects of lower-level exposures that more approximate mTBI by far the most common clinical exposure in the most recent conflicts. There is also little information on whether vascular and inflammatory effects persist chronically. Many of the vascular and inflammatory changes have been documented to occur only transiently. Few studies in animals have addressed changes weeks or months after blast exposure, the phase most relevant to the chronic persistent symptoms that are the most troublesome following human blast-related mTBI.

Yet, despite the paucity of long-term studies, a chronic vascular pathology has been observed in animals. In addition, some blast-related inflammatory effects have been documented to be present weeks or months following exposure. Chronic low-grade inflammation is a consistent feature of many neuropsychiatric disorders including major depression and PTSD. Clearly, a need exists to better understand the nature of vascular damage following blast exposure and the relationship of vascular changes to neuroinflammatory effects. Understanding this relationship will be critical to determining whether chronic immune activation in brain following vascular injury may underlie blast-associated neurobehavioral changes.

## Author Contributions

All authors participated in the collection, review, and analysis of the relevant literature as well as the drafting and revising of the manuscript.

## Conflict of Interest Statement

The authors declare that the research was conducted in the absence of any commercial or financial relationships that could be construed as a potential conflict of interest.
